# Socioeconomic determinants of very frequent presentations to emergency departments in New South Wales, Australia: A state wide data linkage study^☆^

**DOI:** 10.1016/j.heliyon.2024.e36520

**Published:** 2024-08-30

**Authors:** Viola Korczak, Radhika Seimon, Kendall Bein, Stephen Jan, Thomas Lung, Michael Dinh

**Affiliations:** a The George Institute for Global Health, University of New South Wales, Australia; b Emergency Department, Royal Prince Alfred Hospital, Camperdown, Australia; c School of Public Health, University of Sydney, Australia; d RPA Green Light Institute for Emergency Care, Sydney, Australia; e Faculty of Medicine and Health, University of New South Wales, Australia

## Abstract

**Objectives:**

To describe the clinical and longitudinal patterns of presentation, and to understand the underlying socioeconomic characteristics of different modes of presentation.

**Design:**

Retrospective state-wide data linkage analysis of emergency department (ED) presentations. Patients were included if they were 18 years of age or over and presented to the ED over twenty times within any consecutive 365-day period between January 2015 and December 2020. This analysis used routinely collected data from the Emergency Department Data Collection and Admitted Patient Data Collection. The quintile of Socioeconomic Indexes for Area (SEIFA) defined by Australian Bureau of Statistics was used for equity considerations.

**Main outcome measures:**

The main outcomes of interest included patients’ clinical presentation, demographic information and SEIFA score as represented by Index of Relative Socioeconomic Advantage and Disadvantage (IRSAD) quintiles.

**Results:**

There were 417,154 presentations and 5,244 patients who met the inclusion criteria. The majority of the presentations were from SEIFA groups 1 (28.2 %) and 2 (35.6 %). The most common presentations were for drug and alcohol (17.5 %), followed by abdominal pathology (11.8 %) and mental health (11.5 %). In the lowest SEIFA group, the main presenting complaints were for drug and alcohol and administrative presentations. While in the highest SEIFA group, the main presentations were for mental health then abdominal pain, followed by drug and alcohol presentations.

**Conclusion:**

Patients in the lower SEIFA groups tended to be older with lower acuity presentations and were more likely to present to the same facility, more regularly. Patients in the lower SEIFA group were also more likely to present with drug and alcohol and administrative presentations while those in the higher SEIFA groups were more likely to present with mental health presentations. System wide interventions are needed to address the needs of both groups, particularly those from lower socioeconomic backgrounds, who would benefit from improved access to primary care either through access to General Practice or Urgent Care Centres.

## Highlights

• *The known:* There is a cohort of patients who represent frequently to the ED.
• *The new:* The three main causes for presentation were for drug and alcohol, abdominal pathology and mental health presentations. Those in the lowest SEIFA group presented with higher presentations with drug and alcohol, followed by administrative presentations. In the highest SEIFA group, the main presentations were for mental health and abdominal pain.
• *Theimplications:* System wide interventions are needed to address the health needs of patients who present frequently such improving access to afterhours General Practice and Urgent Care Centres in the short term while ensuring drug and alcohol and mental health services are more accessible.

## 1 Introduction

The Australian healthcare system is underpinned by the principle of universal healthcare, though there is increasing inequality in accessing health services in the country [1]. The emergency department (ED) provides a safety net, particularly for those who do not have access to other forms of care in the community. Barriers to accessing care in the community are multiple and include, but are not limited to, knowledge about other health services and costs [2]. Patients who present frequently to the ED often have complex presentations [3,4]. These patients often require multidisciplinary interventions including social work, but such services are often not available after hours when these patients are more likely to present [5]. These issues are likely to disproportionately affect patients from lower socioeconomic backgrounds, for whom private services in the community may be out of reach.

There is no accepted definition for what constitutes a ‘frequent presenter’ [6]. However, in applying an equity lens to address those with the greatest need, the focus should be on those with the highest number of presentations. Dinh et al. (2016), using linked data in New South Wales (NSW) found that patients who present more than 15 times per year represent the top 2.5 % of presentation frequencies [7]. The focus in this paper is on the very frequent presenters, who present more than 20 times per year, which also represents the top 2.5 % of the cohort in this dataset.

Patients who present frequently to the EDs have been identified in multiple countries including Canada [8], Japan [9] and Australia [10,11], among others. A previous study described a snapshot of current practice within NSW hospitals in relation to the presentation and treatment of these patients [12]. The study found that the majority of surveyed clinicians who were working across NSW hospital EDs could identify a cohort of patients who presented frequently in the hospitals in which they worked. A large proportion also had interventions for this group which was largely based around case management [12]. The surveyed clinicians reported a sense of frustration about the lack of co-ordination of care and a lack of resources to improve the health outcomes of this cohort of patients.

The aim of this study is to build on the previous research and further characterise this cohort of patients who present frequently to the ED in NSW, focusing on those who present more than twenty times per year. The aims are to (i) describe the clinical and longitudinal patterns of presentation in this population, and (ii) understand the underlying socioeconomic characteristics and modes of presentation. Doing so, will lead to a better understanding of the needs and specific interventions needed to improve the outcomes.

## 2 Methods

### 2.1 Study setting

New South Wales is the most populated state in Australia. The public health system consists of 228 public hospitals. It serves 8.8 million residents and is the largest public health system in Australia. It consists of 15 local health districts and two specialty networks [13].

### 2.2 Inclusion criteria

This was a retrospective state-wide, data linkage analysis of ED presentations. Patients were included if they were more than 18 years of age and presented to the ED over twenty times within a consecutive 365-day period between January 2015 and December 2020. If at any point a patient had more than 20 presentations in any 365 day period, they were classified as ‘frequent presenters’. All presentations in the study period were collected for all frequent presenters. For example, if patients were identified as frequent presenters in 2019, their presentations from previous years were also included in the analysis. They did not leave the dataset until the end of the study period in December 2020.

### 2.3 Data sources

This analysis used routinely collected data from the Emergency Department Data Collection (EDDC) and Admitted Patient Data Collection (APDC) which contains health and administrative data from all public hospitals in NSW(13). These collections were deterministically linked by the NSW Centre for Health Record Linkage to create an episode of care comprising all relevant ED variables. Data collections were linked by the NSW Centre for Health Record Linkage (CHeReL) using probabilistic record linkage methods, with a range of personal identifiers to cross-sectionally and longitudinally link individuals’ records. Irvine et al. further outlines the linked data process [14].

Socioeconomic Indexes for Areas (SEIFA) indexes from the Australian Bureau of Statistics (ABS) was used to analyse the presentations by quintile and for equity considerations. SEIFA consist of four indexes, and the Index of Relative Socioeconomic Advantage and Disadvantage (IRSAD) was used in this study [15]. The IRSAD is a composite which includes variables such as housing and education and is linked to postcodes. Output is reported in quintiles where ‘one’ denotes greatest area of disadvantage and ‘five’ the greatest area of advantage. SEIFA scores were used as a proxy for socioeconomic status.

### 2.4 Outcome measures

The main outcomes of interest included patients’ clinical presentation, demographic information and SEIFA score as represented by IRSAD. Other variables included variability in time between presentations (temporal clustering index), variability in presenting complaint (diagnostic index) and variability in seeking care at one or multiple health services (facility index). The method for determining the three indexes was developed by coauthor KB and has been published elsewhere [16]. The indexes are outlined below.

The ‘temporal clustering index’ measures the variability in time between presentations. It does not measure the absolute time interval. A low number implies that the patient presents regularly and predictably. Conversely, if a patient presents with time intervals that vary from days to weeks to months, they will have a higher cluster index as there is greater variability in the time interval, and therefore clustering. ‘Diagnostic index’ considers the presenting complaint as described by ICD 9, 10 and Snomed CT codes. If a patient always presents with a single pathology group (for example, respiratory), they score 1. If, however they have more variability in the presenting complaint, the number will be higher. ‘Facility index’ refers to how variable a patient's choice of hospital is. If they always seek care at the one hospital, they will score 1. If, however there is more variability and patients seek care from multiple hospitals, this number will be larger. Peak per year presentation frequency (PPF) represents the maximum number of presentations per patient within a 365-day period.

### 2.5 Statistical analysis

Chi square tests were used to compare categorical variables between SEIFA groups. An analysis of variance (ANOVA) and Wilcoxon rank sum was used to compare median continuous variables. Peak per year presentation frequency, total presentations, admission rate, temporal cluster index, diagnostic index and facility index, were considered to be non-parametric variables. A significance level of 0.05 was used. All statistical analyses were performed using SAS Enterprise Guide (SAS Institute, Cary, NC, USA), Version 9.4.

### 2.6 Ethical approval

Ethics approval was obtained from the NSW Population and Health Services Research Ethics Committee (2019/ETH01600). Informed consent was not required from this study because it was a linked data project with de-identified records.

## 3 Results

In total there were 417,154 presentations between January 2015 and December 2020 in NSW, meeting the study criteria. Table 1 summarises per presentation data, that is all presentations by those who met the inclusion criteria. The majority of presentations were in the bottom two SEIFA quintiles (63.8 %). Triage 5 was the most common triage category (31.6 %), most self-presented (63.6 %) and were subsequently discharged (67.3 %), although a significant proportion were admitted (18.6 %). A further 11.4 % were either discharged against medical advice or did not wait for treatment. The majority of the presentations were after hours (84.5 %), with less than 14.5 % presenting during GP opening hours. The most common ED diagnosis category for all presentations was for drug and alcohol (17.5 %), followed by abdominal pathology (11.8 %), mental health (11.5 %), and administrative (8.3 %) presentations such as for prescriptions **(**Table 1**)**. In the lowest SEIFA group, when excluding the ‘other’ category, the main presenting complaints were for drug and alcohol (19.7 %), administrative issues (13.5 %) and abdominal pain (11 %). In the highest SEIFA group the main presentations were for mental health (18.2 %), abdominal pain (12.8 %) and drug and alcohol presentations (11.9 %).

**Table 1:** Types of presentations for patients presenting more than 20 times per year to NSW ED between 2015 and 2020.

		SEIFA 1	SEIFA 2	SEIFA 3	SEIFA 4	SEIFA 5	Total	P-value
		N (%)	N (%)	N (%)	N (%)	N (%)	N (%)	
Triage	1	405 0.35	618 0.42	361 0.54	531 1.02	424 1.32	2339 0.56	<0.0001
	2	10899 9.28	13509 9.11	8081 12.17	8070 15.53	5134 16.01	45693 10.98	
	3	26996 23.00	35699 24.06	19715 29.69	21824 42.01	12585 39.25	116819 28.07	
	4	26515 22.59	44875 30.25	21484 32.35	16419 31.61	10479 32.68	119772 28.78	
	5	52575 44.79	53659 36.17	16770 25.25	5104 9.83	3445 10.74	131553 31.61	
Total		**117390**	**148360**	**66411**	**51948**	**32067**	**416176**	
Mode of arrival	Ambulance	30547 25.98	43827 29.49	26711 40.10	27973 53.78	16298 50.71	145356 34.86	<0.0001
	Self presented	85516 72.74	102816 69.18	38883 58.37	22907 44.04	15164 47.148	265286 63.62	
	Other	1507 1.28	1975 1.33	1024 1.54	1136 2.18	678 2.11	6230 1.52	
Total		**117570**	**148618**	**66618**	**52016**	**32140**	**416962**	
Mode of separation	Admitted	18254 15.53	23578 15.86	12674 19.02	14412 27.69	8476 26.36	77394 18.56	<0.0001
	Discharged	85296 72.55	108360 72.90	42181 63.30	27303 52.46	17493 54.40	280633 67.29	
	DAMA/DNW*	9827 8.36	13647 9.18	9283 13.93	9384 18.03	5688 17.69	47829 11.47	
	Other	4199 3.57	3063 2.06	2498 3.75	946 1.82	499 1.55	11205 2.69	
Total		**117576**	**148648**	**66636**	**52045**	**32156**	**417061**	
Time of presentation	8am to 5pm	16395 13.94	18917 12.72	10100 15.15	9059 17.40	6136 19.08	60607 14.53	<0.0001
	5pm to midnight	68631 58.35	86128 57.93	34090 51.15	23041 44.26	12888 40.08	224778 53.88	
	Midnight to 8am	32585 27.71	43641 29.35	22456 33.69	19953 38.33	13134 40.84	131769 31.59	
Total		**117611**	**148686**	**66646**	**52053**	**32158**	**417154**	
ED Diagnosis Category	Drug and Alcohol	22005 19.66	30038 21.35	7912 12.78	5129 10.66	3619 11.87	68703 17.48	<0.0001
	Abdominal	12296 10.99	14942 10.62	8693 14.04	6618 13.75	3892 12.77	46441 11.81	
	Mental Health	8453 7.55	13727 9.76	8814 14.23	8666 18.01	5538 18.17	45198 11.50	
	Administrative	15102 13.49	10694 7.60	3724 6.01	1517 3.15	1587 5.21	32624 8.30	
	Injury	7092 6.34	8787 6.25	5193 8.39	3438 7.14	2100 6.89	26610 6.77	
	Cardiovascular	6385 5.71	7821 5.56	4671 7.54	4395 9.13	2294 7.53	25566 6.50	
	MSK	5852 5.23	8116 5.77	3577 5.78	3712 7.71	2083 6.83	23340 5.94	
	Respiratory	6624 5.92	8325 5.92	3008 4.86	2638 5.48	1416 4.65	22011 5.60	
	Neurological	4914 4.39	6585 4.68	3022 4.88	2412 5.01	2192 7.19	19125 4.86	
	Other	23188 20.72	31651 22.50	13310 21.49	9601 19.95	5761 18.90	83511 21.24	
Total		**111911**	**140686**	**61924**	**48126**	**30482**	393129***	

*DAMA/DNW (Discharged Against Medical Advice/Did Not Wait).

** 40801 missing.

MSK: Musculoskeletal.

ED: Emergency Department.

SEIFA: Socio-Economic Indexes for Areas.

Triage 1: seen immediately for life threatening presentations.

Triage 2: seen within 10 min.

Triage 3: within 30 min.

Triage 4: within 1 h.

Triage 5: within 2 h.

Table 2 shows the main diagnoses which were included under each subcategory of the three main presenting complaints. There is more uniformity in the types of presentation in the drug and alcohol cohort and more heterogeneity in the abdominal pain and mental health presentations.

**Table 2:** Three main presenting complaints with subcategories.

Most common presenting complaints	Percentage of total in each category
**Drug and alcohol**	
Drug affected/addicted/use/abuse	74 %
Intoxication/alcohol/abuse	16 %
Overdose/poison/ingestion	6 %
Withdrawal	2 %
Testing	1 %
Other presentations	1 %
**Abdominal pain**	
Abdominal pain/groin/buttock/anorectal	55 %
Gastroenteritis/enteritis/colitis	13 %
Appendix	9 %
Gastritis/haemorrhagic gastritis	6 %
Constipation/faecal impaction	5 %
Other presentations	12 %
**Mental health**	
Disorders/problems/mental health review/mentalhealth/illness assess/observations/consultation	26 %
Depression/low mood	17 %
Schizophrenia/hallucinations/thought disordered/altered/bizarre/disturbed behaviour/delusional/paranoid/psychosis/PTSD/mania	15 %
Aggression/agitated	7 %
Emotional/crisis/upset/stress	7 %
Other presentations	28 %

Table 3 shows per patient data. There were 5,244 patients who presented more than 20 times within a 365-day period. The age at first presentation varied across the group. The largest cohort was between 40 and 59 years (33.9 %). There were more men (55.6 %) than women (44.4 %) who were frequent presenters. Most of the patients spoke English as a first language (91.2 %), though there were 55 languages other than English represented in the sample.

**Table 3:** Per patient data for patients presenting more than 20 times per year to NSW ED between 2015 and 2020.

		SEIFA 1	SEIFA 2	SEIFA 3	SEIFA 4	SEIFA 5	Total	P-value
		N (%)	N (%)	N (%)	N (%)	N (%)	N (%)	
Age at first presentation	<25 years	193 12.69	277 14.92	153 18.84	96 15.34	59 13.79	778 14.84	<0.001
	25–39 years	370 24.33	498 26.82	213 26.23	163 26.04	124 28.97	1368 26.09	
	40–59 years	506 33.27	593 31.93	264 32.51	235 37.54	178 41.59	1776 33.87	
	60–79 years	371 24.39	401 21.59	153 18.84	107 17.09	58 13.55	1090 20.79	
	>80 years	81 5.33	88 4.74	29 3.57	25 3.99	9 2.10	232 4.42	
Total		**1521**	**1857**	**812**	**626**	**428**	**5244**	
Gender	Male	855 56.21	1008 54.28	432 53.20	369 58.95	251 58.64	2915 55.59	0.1
	Female	666 43.79	849 45.72	380 46.80	257 41.05	177 41.36	2329 44.41	
Total		**1521**	**1857**	**812**	**626**	**428**	**5244**	
Language	English	1448 95.20	1708 92.32	772 95.07	540 86.26	313 73.13	4781 91.17	<0.001
	Other	73 4.80	142 7.58	40 4.93	86 13.74	115 26.87	456 8.70	
Total		**1521**	**1850**	**812**	**626**	**428**	**5237**	
		**SEIFA** **Median (IQR)**	**SEIFA** **Median (IQR)**	**SEIFA** **Median (IQR)**	**SEIFA** **Median (IQR)**	**SEIFA** **Median (IQR)**		
Index measures	Peak per year presentation frequency, Median (IQR)	30.0 (25.0–42.0)	30.0 (25.0–43.0)	30.0 (25.0–41.0)	30.0 (25.0–41.0)	30.0 (25.0–43.0)	.	0.95
	Total presentations, Median (IQR)	54.0 (38.0–84.0)	56.0 (38.0–84.0)	62.0 (40.0–91.0)	59.0 (40.0–86.0)	59.0 (40.0–86.0)	.	0.21
	Admission Rate, Median (IQR)	6.0 (2.0–17.0)	7.0 (2.0–17.0)	11.0 (4.0–21.5)	15.0 (7.0–28.0)	14.0 (8.0–26.0)	.	<0.0001
	Temporal Cluster index, Median (IQR)	0.84 (0.56–1.09)	0.85 (0.61–1.19)	0.91 (0.72–1.15)	0.92 (0.73–1.14)	0.94 (0.77–1.16)	.	<0.0001
	Diagnostic index, Median (IQR)	4.71 (2.68–7.28)	4.99 (3.01–7.18)	5.49 (3.62–7.45)	5.59 (3.51–7.68)	5.40 (3.79–7.37)	.	<0.0001
	Facility index, Median (IQR)	1.49 (1.8–2.37)	1.43 (1.19–2.45)	1.73 (1.25–2.95)	2.08 (1.26–3.36)	2.54 (1.60–3.86)	.	<0.0001

IQR: Interquartile range.

SEIFA: Socio-Economic Indexes for Areas.

Index measures summarised in Table 3 show that patients from higher SEIFA groups had higher rates of admission (P < 0.001). The temporal clustering index measuring the variability in the time intervals between presentations showed that the most advantaged group, SEIFA 5 (0.94), had the least variability (P < 0.001)). They were the most likely to present at regular intervals. The diagnostic index was lower in the lower SEIFA groups and slightly higher in the higher SEIFA groups (P < 0.001) indicating that patients from lower socioeconomic backgrounds were more likely to present with the same medical conditions repeatedly, but even then, there was variability in the presenting complaint. The facility index showed that SEIFA 1 was most likely to present repeatedly to the same facility, whereas the most advantaged group was more likely to visit multiple facilities for their treatment (P < 0.001). On the other hand, the patients from lower SEIFA quintiles were more likely to be from rural or regional areas (as SIEFA is linked to rurality as it is based on postcodes). In these areas there are fewer other alternatives and only one hospital that patients can present at, unlike a metropolitan area where patients can present at several different hospitals.

## 4 Discussion

The objective of the paper was to describe the clinical and longitudinal patterns of presentation, and to understand the underlying socioeconomic characteristics and modes of presentation. The two lowest socioeconomic groups, as represented by SEIFA, had the largest number of presentations, accounting for over 60 % of all presentations. The majority of the total presentations were in the two lowest triage categories and patients were most likely to self-present to ED and be discharged. Patients presenting more than 20 times within a 365-day period were more likely to be male and speak English as a first language. The most common ED diagnosis category was for drug and alcohol, followed by abdominal pathology then mental health, although the proportions in each category differed by SEIFA category. Those in the lowest socioeconomic group are more likely to present to the same facility and are also less likely to have variability in their ED diagnosis.

Our findings showed a difference in the way in which patients from higher and lower socioeconomic groups present. While drug and alcohol was the most common ED diagnosis category, the rates of presentation were highest in the lowest SEIFA groups. This correlates with other literature which found that patients who present frequently have higher rates of substance use disorders [17,18]. This may disproportionately affect those within the lowest socioeconomic group who only have limited services as an option [19]. Abdominal pathology was the second most common ED diagnosis and rates of presentations were fairly uniform between the SIEFA groups, but they varied for mental health presentations, which was the third most common ED diagnosis. One reason for this could be that there are few options for patients with mental health presentations. Even when patients have the resources to pay for their care, the ED is usually still the first port of call. This points to broader health system implications of a lack of access to mental health services in the community, especially those offered after hours [20].

Patients in the lowest two SEIFA groups had a high proportion of presentations in the administrative category. These are usually for scripts, medications or wound dressing, for example. These could potentially be treated in the community but access to a general practitioner or the cost of a visit can be prohibitive to patients in the lowest SEIFA. The other barrier is time of visit as these patients are more likely to present out of hours when general practice is not an option. The ED continues to offer a safety net for patients who are unable to access care in the community.

Patients from lower socioeconomic backgrounds tended to have a greater proportion of lower acuity presentations, which may explain the lower rates of admission. They are more likely to present to the same facility and more regularly than higher socioeconomic groups. This is likely due to the fact that, as emphasized in previous literature [21], they are more likely to have more co-morbidities. From the social determinants of health, it is known that non-medical factors such as income, education and employment status, influence health outcomes [22].

### 4.1 Policy implications

Previous discussion around patients who present frequently has focused around decreasing their number and focusing on cost saving measures to the health system. The main driver for policy interventions and discussions has been focussed on health system drivers [23]. These data show that adopting an ‘equity’ approach to policy interventions, targeting those who are most in need of additional services, may be more beneficial.

The study showed that there are two distinct groups in the frequent presenter cohort; those from lower socioeconomic backgrounds who are generally older and present with lower acuity presentations who had high drug and alcohol and administrative presentations, and those from a higher socioeconomic background who have a greater proportion of mental health presentations. A one size approach is unlikely to work, rather targeted interventions should be used to optimise outcomes.

Patients in the lower SEIFA groups were presenting with less clustering and at more regular intervals (lower temporal cluster index) and were more likely to present with the same presenting compliant (as determined by the diagnostic index). Whereas the higher SEIFA groups had more variability in temporal and diagnostic clustering with less predictable patterns of presentation. The more predictable patterns in presentation in the lower SEIFA group makes them more amenable to policy interventions.

Lower SEIFA groups would benefit from improved access to primary care. This highlights tensions between care provision with primary care funded largely by the Commonwealth and hospital-based care which is funded mostly by state governments. There is a disconnect between health services and how they are funded which may impact access to healthcare in the community, especially in rural areas. Improving access to afterhours General Practice and public Urgent Care Centres may especially apply to low acuity frequent presenters in rural and regional locations.

Another example of a targeted approach for those in the lower SEIFA groups is case management. One such example is the ‘ED to Community’ program currently being trialled through the Sydney Local Health District [24] which provides multidisciplinary care to patients with complex care needs.

For those patients from the higher SEIFA group who present with mental health presentations, additional services should be available in the community. While NSW Health has outlined a clear mental health strategy [25], current services are not meeting demand. With the rising number of mental health presentations, especially post covid [26], a strategic ‘rethink’ around policy on community mental health is needed.

### 4.2 Strengths and limitations

A strength of the study was the large, linked data set which included presentations to the ED across NSW. The data also allowed for the patients to be followed longitudinally. A limitation was the method used to analyse the data is novel and its utility is not well described. However, it has been used previously in a published study [16]. The method has found significance and correlates in both papers, thus supporting its validity.

## 5 Conclusions

In the lowest SEIFA group the main presenting complaints are for drug and alcohol and administrative presentations, whereas in the highest SEIFA group the main presentations were for mental health and abdominal pain. The policy debate needs to change from one focussed on reducing the number of ‘frequent presenters’ to one around equity-based healthcare delivery, which is truly focused on universal health outcomes and addresses the specific needs of individual patients. Policy should focus on increasing access to afterhours primary care, especially addressing the main presenting complaints in the lowest SEIFA group; drug and alcohol and administrative presentations.

## Data availability statement

Data subject to third party restrictions. The data that support the findings of this study are not publicly available due to privacy or ethical restrictions.

## CRediT authorship contribution statement

**Viola Korczak:** Writing – review & editing, Writing – original draft, Methodology, Formal analysis, Conceptualization. **Radhika Seimon:** Writing – review & editing, Methodology, Formal analysis, Data curation. **Kendall Bein:** Writing – review & editing, Methodology, Data curation, Conceptualization. **Stephen Jan:** Writing – review & editing, Conceptualization. **Thomas Lung:** Writing – review & editing, Conceptualization. **Michael Dinh:** Writing – review & editing, Supervision, Methodology, Formal analysis, Data curation, Conceptualization.

## Declaration of competing interest

The authors declare that they have no known competing financial interests or personal relationships that could have appeared to influence the work reported in this paper.

## References

[1] Zuccala E. (2023). What is the future of universal health coverage in Australia?. Med. J. Aust..

[2] Australian Institute of Health and Welfare (2016).

[3] Sun B.C., Burstin H.R., Brennan T.A. (2003). Predictors and outcomes of frequent emergency department users. Acad. Emerg. Med. : official journal of the Society for Academic Emergency Medicine.

[4] Binnie V., Le Brocque R., Jessup M., Johnston A.N.B. (2021). Adult frequent presentation to emergency departments and adverse childhood experiences: a scoping review. Australas Emerg Care.

[5] Birmingham L.E., Cochran T., Frey J.A., Stiffler K.A., Wilber S.T. (2017). Emergency department use and barriers to wellness: a survey of emergency department frequent users. BMC Emerg. Med..

[6] Korczak V., Shanthosh J., Jan S., Dinh M., Lung T. (2019). Costs and effects of interventions targeting frequent presenters to the emergency department: a systematic and narrative review. BMC Emerg. Med..

[7] Dinh M.M., Berendsen Russell S., Bein K.J., Chalkley D., Muscatello D., Paoloni R. (2016). Trends and characteristics of short-term and frequent representations to emergency departments: a population-based study from New South Wales, Australia. Emerg. Med. Australasia (EMA) : Emerg. Med. Australasia (EMA).

[8] Moe J., Wang E.Y., McGregor M.J., Schull M.J., Dong K., Holroyd B.R. (2022). People who make frequent emergency department visits based on persistence of frequent use in Ontario and Alberta: a retrospective cohort study. CMAJ Open.

[9] Takeuchi S., Funakoshi H., Nakashima Y., Homma Y., Takahashi J., Shiga T. (2019). Unique characteristics of frequent presenters to the emergency department in a Japanese population: a retrospective analysis. Acute Med Surg.

[10] Cordell B., Nguyen K., Shahir A.K., Lord S.J., Gallego G. (2022). Prevalence and characteristics of frequent presenters to Auburn Hospital emergency department. Emerg. Med. Australasia (EMA) : Emerg. Med. Australasia (EMA).

[11] Markham D., Graudins A. (2011). Characteristics of frequent emergency department presenters to an Australian emergency medicine network. BMC Emerg. Med..

[12] Korczak V., Liu H., Bein K., Lung T., Jan S., Dinh M. (2023). Emergency clinician perceptions of patients who present frequently to the emergency department: a snapshot of current practice. Aust. Health Rev. : a publication of the Australian Hospital Association.

[13] New South Wales Health (2023).

[14] Irvine K., Hall R., Taylor L. (2019). A profile of the centre for health record linkage. Int J Popul Data Sci..

[15] Australian Bureau of Statistics (2023). https://www.abs.gov.au.

[16] Stanford D., Dinh M.M., Eastwood J.G., Korczak V., Seimon R.V., Moore C. (2024). Clinical and longitudinal patterns of frequent presenters to emergency departments: a multi-centre data linkage analysis. Emerg. Med. Australasia (EMA) : Emerg. Med. Australasia (EMA).

[17] Curran G.M., Sullivan G., Williams K., Han X., Allee E., Kotrla K.J. (2008). The association of psychiatric comorbidity and use of the emergency department among persons with substance use disorders: an observational cohort study. BMC Emerg. Med..

[18] Locker T.E., Baston S., Mason S.M., Nicholl J. (2007). Defining frequent use of an urban emergency department. Emerg. Med. J. : Eng. Manag. J..

[19] McIntyre H., Reeves V., Loughhead M., Hayes L., Procter N. (2022). Communication pathways from the emergency department to community mental health services: a systematic review. Int. J. Ment. Health Nurs..

[20] Sacre M., Albert R., Hoe J. (2022). What are the experiences and the perceptions of service users attending Emergency Department for a mental health crisis? A systematic review. Int. J. Ment. Health Nurs..

[21] Hansagi H., Allebeck P., Edhag O. (1989). Health care utilization after referral from a hospital emergency department. Scand. J. Soc. Med..

[22] Bush M. (2018). Addressing the root cause: rising health care costs and social determinants of health. N. C. Med. J..

[23] Lowthian J.A., Curtis A.J., Cameron P.A., Stoelwinder J.U., Cooke M.W., McNeil J.J. (2011). Systematic review of trends in emergency department attendances: an Australian perspective. Emerg. Med. J. : Eng. Manag. J..

[24] RPA Virtual (2022).

[25] Mental Health Commission of New South Wales (2014).

[26] Ren F.F., Guo R.J. (2020). Public mental health in post-COVID-19 era. Psychiatr. Danub..

