# Supplementary material for: Socioeconomic determinants of very frequent presentations to emergency departments in New South Wales, Australia: A state wide data linkage study
Source: Heliyon. 2024 Aug 30;10(17):e36520. doi: 10.1016/j.heliyon.2024.e36520 (PMC11402644; doi:10.1016/j.heliyon.2024.e36520)
Supplement: Multimedia component 1 [file mmc1.pdf]

STROBE Statement—checklist of items that should be included in reports of observational studies

	Item No	Recommendation	Page No
**Title and abstract**	1	(*a*) Indicate the study's design with a commonly used term in the title or the abstract	Pg 1 line 1
		(*b*) Provide in the abstract an informative and balanced summary of what was done and what was found	Pg 2 line 44
**Introduction**			
Background/rationale	2	Explain the scientific background and rationale for the investigation being reported	Pg 4 line 108
Objectives	3	State specific objectives, including any prespecified hypotheses	Pg 4, line 128
**Methods**			
Study design	4	Present key elements of study design early in the paper	Pg 5, line 135
Setting	5	Describe the setting, locations, and relevant dates, including periods of recruitment, exposure, follow-up, and data collection	Pg 5, line 134
Participants	6	(*a*) *Cohort study*—Give the eligibility criteria, and the sources and methods of selection of participants. Describe methods of follow-up<br>*Case-control study*—Give the eligibility criteria, and the sources and methods of case ascertainment and control selection. Give the rationale for the choice of cases and controls<br>*Cross-sectional study*—Give the eligibility criteria, and the sources and methods of selection of participants	—
		(*b*) *Cohort study*—For matched studies, give matching criteria and number of exposed and unexposed<br>*Case-control study*—For matched studies, give matching criteria and the number of controls per case	—
Variables	7	Clearly define all outcomes, exposures, predictors, potential confounders, and effect modifiers. Give diagnostic criteria, if applicable	Pg 6 line 175
Data sources/ measurement	8*	For each variable of interest, give sources of data and details of methods of assessment (measurement). Describe comparability of assessment methods if there is more than one group	Pg 5 line 150
Bias	9	Describe any efforts to address potential sources of bias	Pg 5, line 150
Study size	10	Explain how the study size was arrived at	Pg 5, 140
Quantitative variables	11	Explain how quantitative variables were handled in the analyses. If applicable, describe which groupings were chosen and why	Pg 6, line 188
Statistical methods	12	(*a*) Describe all statistical methods, including those used to control for confounding	Pg 6 line 167
		(*b*) Describe any methods used to examine subgroups and interactions	Pg 6, line 170
		(*c*) Explain how missing data were addressed	Pg 6, line 175
		(*d*) *Cohort study*—If applicable, explain how loss to follow-up was addressed<br>*Case-control study*—If applicable, explain how matching of cases and controls was addressed<br>*Cross-sectional study*—If applicable, describe analytical methods taking account of sampling strategy	—
		(*e*) Describe any sensitivity analyses	—

Continued on next page

**Results**			
Participants	13*	(a) Report numbers of individuals at each stage of study—eg numbers potentially eligible, examined for eligibility, confirmed eligible, included in the study, completing follow-up, and analysed	Pg 7 line 202
		(b) Give reasons for non-participation at each stage	—
		(c) Consider use of a flow diagram	—
Descriptive data	14*	(a) Give characteristics of study participants (eg demographic, clinical, social) and information on exposures and potential confounders	Pg 7 line 223
		(b) Indicate number of participants with missing data for each variable of interest	—
		(c) *Cohort study*—Summarise follow-up time (eg, average and total amount)	—
Outcome data	15*	*Cohort study*—Report numbers of outcome events or summary measures over time	—
		Case-control study—Report numbers in each exposure category, or summary measures of exposure	—
		Cross-sectional study—Report numbers of outcome events or summary measures	—
Main results	16	(*a*) Give unadjusted estimates and, if applicable, confounder-adjusted estimates and their precision (eg, 95% confidence interval). Make clear which confounders were adjusted for and why they were included	—
		(*b*) Report category boundaries when continuous variables were categorized	—
		(*c*) If relevant, consider translating estimates of relative risk into absolute risk for a meaningful time period	—
Other analyses	17	Report other analyses done—eg analyses of subgroups and interactions, and sensitivity analyses	Pg 8 line 230
**Discussion**			
Key results	18	Summarise key results with reference to study objectives	Pg 8, line 244
Limitations	19	Discuss limitations of the study, taking into account sources of potential bias or imprecision. Discuss both direction and magnitude of any potential bias	Pg 11, line 326
Interpretation	20	Give a cautious overall interpretation of results considering objectives, limitations, multiplicity of analyses, results from similar studies, and other relevant evidence	Pg 11, line 333
Generalisability	21	Discuss the generalisability (external validity) of the study results	Pg 11, line 334
**Other information**			
Funding	22	Give the source of funding and the role of the funders for the present study and, if applicable, for the original study on which the present article is based	Pg 1 line 17

*Give information separately for cases and controls in case-control studies and, if applicable, for exposed and unexposed groups in cohort and cross-sectional studies.

Note: An Explanation and Elaboration article discusses each checklist item and gives methodological background and published examples of transparent reporting. The STROBE checklist is best used in conjunction with this article (freely available on the Web sites of PLoS Medicine at http://www.plosmedicine.org/, Annals of Internal Medicine at http://www.annals.org/, and Epidemiology at http://www.epidem.com/). Information on the STROBE Initiative is available at www.strobe-statement.org.
